# Brodalumab: Six-Year US Pharmacovigilance Report

**DOI:** 10.1007/s13555-024-01304-y

**Published:** 2024-11-26

**Authors:** Mark G. Lebwohl, John Y. Koo, April W. Armstrong, Bruce E. Strober, Soo Han Yoon, Nicole N. Rawnsley, Earl L. Goehring, Gina D. Mangin, Abby A. Jacobson

**Affiliations:** 1 Icahn School of Medicine at Mount Sinai, 5 East 98th Street, 5th Floor, New York, NY 10029 USA; 2 Psoriasis and Skin Treatment Center, University of California, San Francisco, San Francisco, CA USA; 3 University of California, Los Angeles, Los Angeles, CA USA; 4 Central Connecticut Dermatology Research, Cromwell, CT USA; 5 Yale University, New Haven, CT USA; 6 Salix Pharmaceuticals, Bridgewater, NJ USA; 7 Bausch Health Companies Inc, Bridgewater, NJ USA; 8 Sand Lake Dermatology Center, Orlando, FL USA

**Keywords:** Adverse events, Drug reaction, Psoriasis, Real-world, Safety

## Abstract

**Introduction:**

Brodalumab is a human interleukin-17 receptor A antagonist indicated for the treatment of moderate-to-severe plaque psoriasis in adult patients who are candidates for systemic therapy or phototherapy and have failed to respond or have lost response to other systemic therapies. In the USA, brodalumab has a boxed warning regarding suicidal ideation and behavior and is only available under a Risk Evaluation and Mitigation Strategy, but no causal association has been established. To assess long-term safety of brodalumab, we summarize pharmacovigilance data from 6 years of real-world clinical practice.

**Methods:**

Crude adverse event (AE) reporting rates per 100 patients were calculated for common AEs and AEs of special interest reported to Ortho Dermatologics by US patients and healthcare providers from August 15, 2017 through August 14, 2023. Brodalumab exposure was estimated as time from the first to last prescription-dispensing authorization dates. Adverse events were defined by Medical Dictionary for Regulatory Activities v26.0 Preferred Terms and standardized MedDRA queries.

**Results:**

Data were collected from 5138 US patients (estimated exposure of 6900 patient-years). Over 6 years, 13 cases of adjudicated major adverse cardiovascular events were reported (0.25 events/100 patients). The rate of serious infections was 2.20 events/100 patients. Since the 5-year report, there was one new case of *Candida* infection and a serious fungal infection of the elbow. Among 57 reported malignancies affecting 49 patients, 4 were deemed possibly related to brodalumab. One new case of indeterminate inflammatory bowel disease unrelated to brodalumab was reported. No new suicide attempts were reported in year 6, and there were no completed suicides throughout 6 years.

**Conclusion:**

Pharmacovigilance data throughout 6 years are consistent with the safety profile of brodalumab established in clinical trials and previous US pharmacovigilance reports, with no completed suicides and a low fungal infection rate.

**Graphical Abstract:**

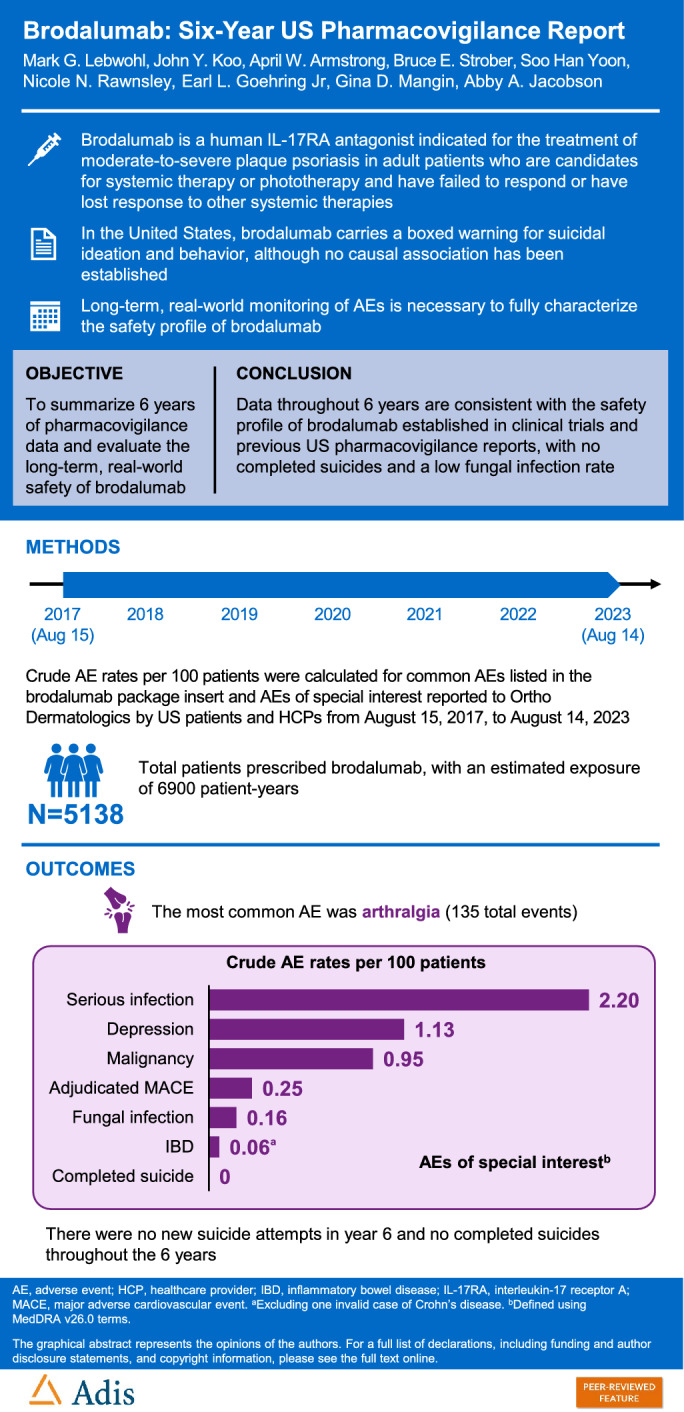

## Plain Language Summary

Brodalumab is an injectable treatment approved for moderate-to-severe plaque psoriasis in adults who lacked response to previous treatments. In the USA, brodalumab is only available under a Risk Evaluation and Mitigation Strategy for increased suicidality risks; however, findings from 6 years of real-world safety data have demonstrated a lack of association. In this report, we discuss safety findings reported by US patients and healthcare providers for 5138 patients treated with brodalumab over 6 years. Joint pain (known as arthralgia) was the most common safety finding, with 135 cases reported over 6 years. Other safety findings of interest across 6 years included 113 cases of serious infection (defined as prolonged infection or infection requiring intervention), 58 cases of depression, 57 cases of cancer (in 49 patients), 56 confirmed cases of COVID-19, and 13 cases of major cardiovascular events (such as heart attack or stroke). No completed suicides occurred throughout 6 years, and no new suicide attempts were reported in year 6. In indirect comparisons with safety data from patients with psoriasis receiving or eligible to receive similar treatments, brodalumab was not associated with an increased risk of serious infection, cancer, major cardiovascular events, or inflammatory bowel disease. Taken together, these data are consistent with safety findings from long-term clinical trials and previous safety reports of brodalumab.

## Key Summary Points

**Table:** 

***Why carry out the study?***
Brodalumab, a human interleukin-17 receptor A antagonist indicated for treatment of moderate-to-severe plaque psoriasis in adults, carries a boxed warning in the US prescribing information regarding suicidal ideation and behavior.
Because patients with psoriasis often require long-term treatment, ongoing monitoring of adverse events (AEs) is necessary to establish the safety profile of brodalumab in a real-world setting.
This report summarizes 6 years (August 15, 2017 through August 14, 2023) of brodalumab pharmacovigilance data reported to Ortho Dermatologics by US patients and healthcare providers with a focus on the most common AEs listed in the brodalumab package insert and AEs of special interest, including suicide and depression.
***What was learned from the study?***
The most commonly reported AE was arthralgia (135 total events); no new suicide attempts were reported in year 6, and no completed suicides were reported throughout the 6 years.
This 6-year pharmacovigilance report supports the consistent safety profile of brodalumab established in pivotal trials and previous pharmacovigilance reports, with no new occurrences of completed or attempted suicide and a consistently low fungal infection rate.

## Digital Features

This article is published with digital features, including a graphical abstract to facilitate understanding of the article. To view digital features for this article, go to 10.6084/m9.figshare.27247380.

## Introduction

Brodalumab is a human anti-interleukin (IL)−17 receptor A monoclonal antibody indicated for the treatment of moderate-to-severe plaque psoriasis in adults [1]. The unique mechanism of action of brodalumab allows for blockade of multiple IL-17 isoforms that contribute to psoriatic disease, including IL-17A, IL-17C, and IL-17F; treatment with brodalumab also reduces the expression of IL-23 and IL-12 subunit genes, indicating that brodalumab may reduce inflammatory markers of psoriasis upstream of IL-17 receptor A [2–4].

Pharmacovigilance reports for the past 5 years have provided real-world evidence to fully characterize the safety profile of brodalumab, which was initially assessed in one phase 2 and two phase 3 studies [5–14]. In the USA, brodalumab carries a boxed warning in the prescribing information regarding suicidal ideation and behavior and is only available under a Risk Evaluation and Mitigation Strategy (REMS); however, no established causal relationship between brodalumab and suicidality has been established during pivotal trials or subsequent pharmacovigilance reports [1, 15]. Moreover, there were no specific exclusion criteria in brodalumab clinical trials regarding history of psychiatric disorders, suicidality, known suicidality risk factors, or substance abuse diagnoses [15].

Along with clinical trials, real-world data allow for a more robust assessment of long-term safety concerns and can help fully characterize the safety profile of brodalumab. In the 5-year US pharmacovigilance report of brodalumab, we highlighted its consistency with the established safety profile reported in long-term clinical trials and previous pharmacovigilance reports [14]. In the current report, we detail 6-year pharmacovigilance data in the USA as an update to the 5-year report.

## Methods

Methods have been previously described [7–9, 12, 14]. Briefly, in this analysis, we summarize pharmacovigilance data reported by US patients and healthcare providers (HCPs) to Ortho Dermatologics (a division of Bausch Health Companies Inc), which markets brodalumab in the USA. Data are from August 15, 2017 through August 14, 2023. Adverse events (AEs) were defined by Medical Dictionary for Regulatory Activities (MedDRA) v26.0 Preferred Terms (PTs) and standardized MedDRA queries. The prevalence of the most common AEs listed in the brodalumab package insert (i.e., AEs with an incidence ≥ 1%) [1] and other AEs of special interest are summarized with descriptive statistics.

Overall drug exposure was estimated as the time between the initial and last prescription-dispensing authorization dates. Detailed medical histories, previous psoriatic therapies, time intervals between previous therapy and brodalumab initiation, and AE dates were either missing or not included in pharmacovigilance reports [8]. Thus, crude AE reporting rates per 100 patients are reported here.

Ethics approval and informed consent were not required, as the postmarketing data summarized in this report were noninterventional and were not collected as part of a clinical study. The collected data were nonidentifiable.

## Results

### Exposure and Unique Cases

Within the 6-year period, data were collected from 5138 US patients who were administered brodalumab, with an estimated exposure of 6900 patient-years. Of the 5138 US patients, 2553 (50%) had ≥ 1 AE reported; 24% were reported by HCPs, and 76% were reported by patients.

### Common AEs

In the brodalumab package insert, common AEs listed include arthralgia, headache, fatigue, diarrhea, oropharyngeal pain, nausea, myalgia, injection-site reactions, influenza, neutropenia, and *Tinea* infections [1]. During the 6-year pharmacovigilance period, arthralgia was the most commonly reported AE, as previously reported in the 5-year report [14]. There were 13 new cases of arthralgia (135 total events) since the 5-year report, resulting in similar crude AE reporting rates of 2.57 events/100 patients in year 5 and 2.63 events/100 patients in year 6. Since the 5-year report, there were seven new cases of headache (1.03 events/100 patients), five new cases of myalgia (0.74 events/100 patients), five new cases of fatigue (0.97 events/100 patients), three new cases of injection-site reactions (0.80 events/100 patients), and two new cases of diarrhea (0.68 events/100 patients). There was one new case each of oropharyngeal pain (0.45 events/100 patients), nausea (0.58 events/100 patients), and influenza (0.47 events/100 patients) in year 6. There were no new cases of neutropenia (0.02 events/100 patients). In the USA, no *Tinea* infections were reported throughout 6 years.

### Other AEs of Special Interest

**Fig. 1:**
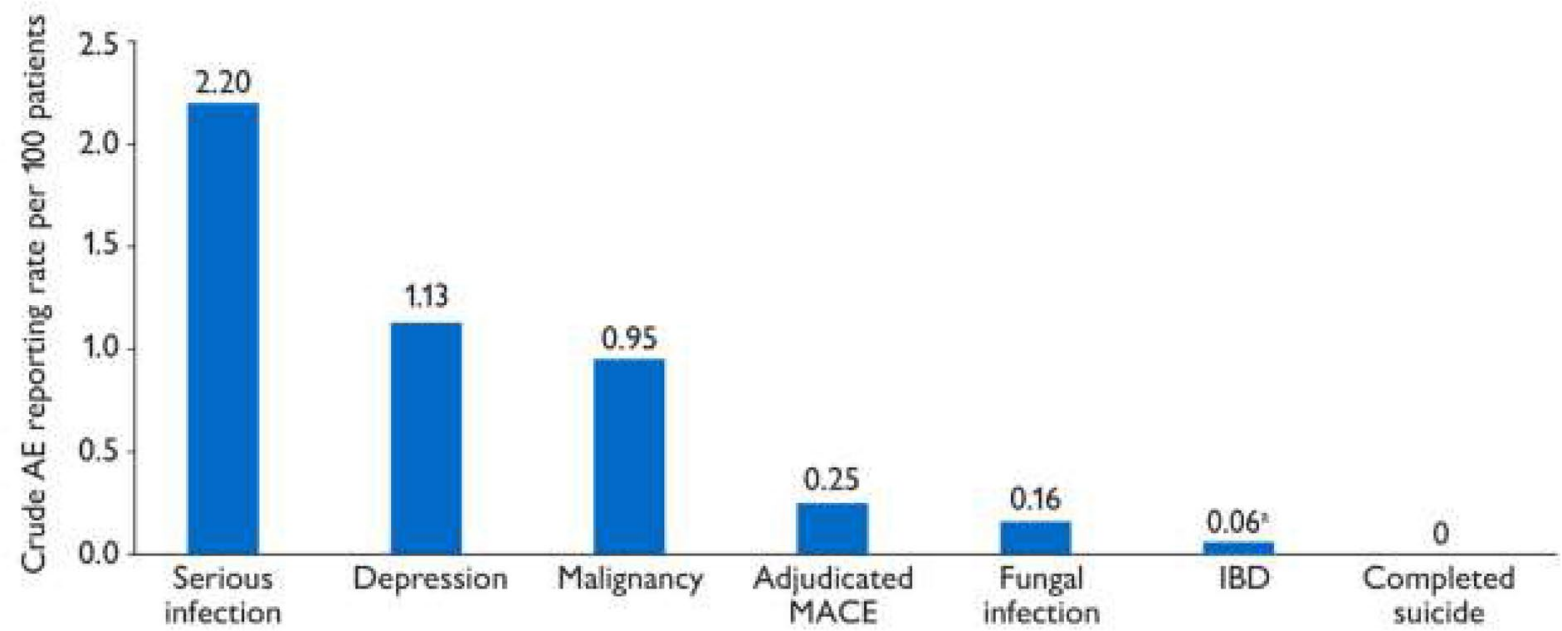
Crude AE reporting rates of clinical events of special interest, as deemed by the reporter or company, per 100 patients. Crude AE reporting rate per 100 patients is the number of clinical events of special interest divided by 5138 brodalumab patients multiplied by 100 patients. Completed suicide events (*n* = 0) are reported. Clinical events of special interest were defined using MedDRA v26.0 terms. Serious infection (i.e., prolonged infection or infection requiring intervention) included any MedDRA PT identified under the SOC name *Infections and infestations*, for which the SOC was indicated as “primary” and event seriousness as “serious.” Depression included MedDRA PTs from *Depression* (excluding *Suicide* and *Self-injury*) SMQ (narrow). Malignancy included MedDRA PTs from *Malignancies* SMQ and *Malignant lymphomas* SMQ. Adjudicated MACE included MedDRA PTs from *Ischemic CNS vascular conditions* SMQ, *Ischemic heart disease* SMQ (*Myocardial infarction*), and *Ischemic heart disease* SMQ (*Other*), and where “medically confirmed” was blank. Fungal infection included MedDRA HLTs from *Fungal infections* NEC. Inflammatory bowel disease included MedDRA PTs *Inflammatory bowel disease*, *Crohn’s disease*, and *Colitis ulcerative*. *AE* adverse event, *CNS* central nervous system, *HLT* high-level term, *IBD* inflammatory bowel disease, *MACE* major adverse cardiovascular events, *MedDRA v26.0* Medical Dictionary for Regulatory Activities, version 26.0, *NEC* not elsewhere classified, *PT* preferred term, *SMQ* standardized MedDRA query, *SOC* system organ class. ^a^Excluding one invalid case of Crohn’s disease

Other AEs that have been deemed of special interest by the reporter or company include adjudicated major adverse cardiovascular events (MACE), serious infections, fungal infections, inflammatory bowel disease (IBD), malignancy, depression, and completed suicide (Fig. 1).

#### Adjudicated MACE

Adjudicated MACE were medically confirmed by the reporting HCP. The crude AE reporting rate of adjudicated MACE for the 6-year period (0.25 events/100 patients) was lower than that of the Psoriasis Longitudinal Assessment and Registry (PSOLAR; 1.55 events/100 patients), an international registry that enrolled 12,095 patients with psoriasis who were receiving or eligible to receive systemic or biologic therapy, with an estimated follow-up of 31,818 patient-years [16]. Throughout the 6 years, a total of 13 cases of adjudicated MACE were reported: 5 cases of myocardial infarction and 1 case each of stroke, ischemic stroke, acute coronary syndrome, left carotid stenosis, unstable angina, coronary artery bypass procedure, coronary artery disease requiring a stent, and transient ischemic attack. For 12 (92%) of these cases, patients had a known history of cardiovascular-associated risk factors (e.g., smoking, obesity), diagnoses/complications (e.g., hypertension, diabetes), or concomitant medications (e.g., aspirin, warfarin, metoprolol). Seven (54%) patients were reported to have continued brodalumab after the event.

#### Infections

Serious infections (i.e., prolonged infections or infections requiring intervention) were defined as any MedDRA v26.0 PT identified under the System Organ Class (SOC) name “Infections and infestations,” for which the SOC was indicated as “primary” and event seriousness as “serious.” Throughout the 6 years, there were 113 cases of serious infections, 4 (4%) of which were determined to be related to brodalumab. The rate of serious infections (2.20 events/100 patients) slightly decreased compared with the 5-year report (2.23 events/100 patients) [14]. There was one new case of *Candida* infection since the 5-year report; this patient continued brodalumab. Additionally, there was one new serious fungal infection of the elbow after surgery, and this patient discontinued brodalumab; for this case, the reporter did not report causality, and the company reported possible causality. There were no new cases of hepatitis or tuberculosis (including latent infection or reactivation) compared with the 5-year reporting period [14].

Since the beginning of the pandemic, there were 56 confirmed and 2 suspected cases of coronavirus disease 2019 (COVID-19). Among these cases, 41 (73%) and 13 (23%) had underlying comorbid conditions or no information regarding comorbidities, respectively. There were no new deaths due to COVID-19 in years 5 and 6 [14].

#### Inflammatory Bowel Disease

Throughout 6 years, three cases of inflammatory bowel disease were reported (0.06 events/100 patients), including one new case of indeterminate inflammatory bowel disease reported in a patient with a history of diarrhea. The patient discontinued treatment, although the investigator did not attribute the event to brodalumab. The patient rated general well-being as “very well” and abdominal pain as mild.

#### Malignancy

The crude AE reporting rate for malignancy in year 6 (0.95 events/100 patients; 57 malignancies in 49 cases) was similar to that in year 5 (0.89 events/100 patients; 49 malignancies in 42 cases) [14] and less than that in PSOLAR (1.78 events/100 patients [excluding nonmelanoma skin cancer]) [16]. Types of reported malignancies in new cases included dermatologic malignancies, such as nonmelanoma skin cancer (both basal cell carcinoma and squamous cell carcinoma) and malignant melanoma; invasive ductal breast carcinoma (patient underwent right breast lumpectomy); and lung and breast cancers. In one case, both basal cell carcinoma (recurrence of malignancy) and malignant melanoma were reported. Of the seven new cases, three patients continued, and four patients discontinued brodalumab. Four (8%) of the reported malignancies throughout the 6 years of data were deemed possibly related to brodalumab (keratoacanthoma-type squamous cell carcinoma, basal cell carcinoma, unspecified neoplasm [carcinoma removed from left hip], and breast cancer).

#### Depression and Previous Case of Suicide Attempt

Throughout 6 years, there were 58 documented cases of depression, with a crude AE reporting rate slightly lower than that of the 5-year report (1.13 vs 1.14 events/100 patients) [14]. The 4 (7%) cases deemed as related to brodalumab all occurred before the 4-year pharmacovigilance report [7, 12]. Of the four new cases in year 6, reporters did not provide causality assessments. No new suicide attempts were reported in year 6, one suicide attempt was previously reported in year 3 [7], and no completed suicides were reported throughout the 6 years.

## Discussion

This US pharmacovigilance report summarizes 6 years of the most common AEs from the brodalumab package insert and additional AEs of special interest reported from August 15, 2017 through August 14, 2023. Consistent with clinical trials and previous pharmacovigilance reports, brodalumab exhibited a tolerable safety profile with no new safety signals reported. There were no new reports of neutropenia, and, in the USA, no *Tinea* infections have been reported throughout the 6 years. Among 13 cases of adjudicated MACE, most patients (92%) had a history of cardiovascular-associated risk factors, diagnoses/complications, or concomitant medications. Across 6 years, there were 56 cases of confirmed COVID-19 and 2 cases of suspected COVID-19; there were no COVID-19-related deaths during year 6. Most patients with COVID-19 (73%) had underlying comorbid conditions. Among 58 documented cases of depression, 4 (7%) were reported to be related to brodalumab (as described in the 3- and 4-year pharmacovigilance reports) [7, 12]. No new cases of suicide attempts were reported during year 6, and no completed suicides were reported throughout the 6 years.

To contextualize crude rates of AEs of special interest reported here, we conducted indirect comparisons with those of other recent observational studies. PSOLAR was an intercontinental registry including 12,095 patients with psoriasis receiving or eligible to receive biologic or systemic therapy (median follow-up, 2.5 years) [16]. Crude rates reported in this 6-year report compared with those calculated from PSOLAR were lower for adjudicated MACE (0.25 vs 1.55 events/100 patients), serious infections (2.20 vs 3.95 events/100 patients), and malignancy (0.95 vs 1.78 events/100 patients [excluding nonmelanoma skin cancer in PSOLAR]). The crude rate of IBD observed here was the same as that from the 5-year report (0.06 events/100 patients for both) and lower than the rate previously calculated from a 20-year Danish study, which included a cohort of 235,038 patients with psoriasis receiving topical, systemic, or biologic therapy [14, 17]. The crude rates of definite Crohn’s disease and/or ulcerative colitis were 0.32 events/100 patients among those receiving any therapy (mean follow-up, approximately 6.8 years) and 0.14 events/100 patients among those receiving biologic therapy (mean follow-up, approximately 3.7 years). Because the prescribing information for brodalumab lists Crohn’s disease as a contraindication [1], the crude rate of IBD reported here may therefore be lower than that observed in other broader populations with psoriasis; however, this possibility is unlikely, as the Danish study excluded patients with a history of confirmed or suspected IBD to ensure that only new-onset cases were captured [17]. Thus, brodalumab is not associated with a heightened risk of adjudicated MACE, serious infection, malignancy, or IBD compared with other systemic or biologic therapies. However, results from both indirect comparisons have limited interpretability due to various factors, including a lack of objective controls and potentially different diagnostic criteria for AEs of special interest.

There are several limitations related to the nature of pharmacovigilance reporting. For instance, only AEs reported to Ortho Dermatologics were documented, and the lack of controlled comparison groups hinders the interpretation of findings. Furthermore, because exact brodalumab administration dates are not available, patient-exposure estimates are based on prescription-dispensing authorization dates. Additionally, a lack of contextual information (typical in data reported via pharmacovigilance channels) restricts our interpretation of the association between brodalumab and reported AEs. Lastly, because brodalumab is only available through a restricted REMS program in the USA [1], treatment is not likely to be prescribed to patients with a known history of depression or suicidal ideation and behavior. Thus, it could be argued that the patient population in this report may not be representative of a real-world distribution of depression. However, a real-world analysis of patients with psoriasis initiating biologic therapy at or after enrollment in the CorEvitas (formerly Corrona) Psoriasis Patient Registry showed that prevalence rates of history of depression were similar among those initiating brodalumab, IL-12/23 or IL-23 inhibitors, or non-brodalumab IL-17A inhibitors (23%, 26%, and 27%, respectively) [18]. These findings suggest that despite the REMS-based program restrictions, rates of depression observed in this pharmacovigilance report may indeed reflect those of a real-world population. Regardless of potential selection biases, if a treatment were associated with a particular AE, it may be expected to occur within both screened and age-matched nonscreened populations. Further, global postmarketing data on brodalumab suggest the risk of suicide is comparable to that of other common biologics [19]. In the opinion of the physician authors of this paper, currently available real-world data do not support that brodalumab poses a unique or increased risk of suicide compared with similar biologics.

## Conclusion

When selecting an optimal therapy for psoriasis, HCPs and patients must balance patient-specific factors, efficacy, and risk of AEs [20–22]. This 6-year US pharmacovigilance report supports the consistent safety profile of brodalumab established in pivotal trials and previous pharmacovigilance reports. Notably, no new cases of suicide attempts were reported in year 6, and no completed suicides were reported throughout the 6 years in the USA. Overall, brodalumab was well tolerated, with no new safety signals emerging after 6 years of pharmacovigilance monitoring, and a consistently low fungal infection rate.

## Data Availability

The datasets generated or analyzed during the current study are available from the corresponding author on reasonable request.
